# Hybrid Odontogenic Lesions: A 30-year Retrospective Study 

**DOI:** 10.30476/dentjods.2024.102367.2358

**Published:** 2025-09-01

**Authors:** Saede Atarbashi-Moghadam, Termeh Sarrafan Sadeghi, Shokoufeh Shahrabi-Farahani, Leyla Roghanizadeh, Sanaz Gholami Toghchi

**Affiliations:** 1 Dept. of Oral and Maxillofacial Pathology, School of Dentistry, Shahid Beheshti University of Medical Sciences, Tehran, Iran.; 2 Dental Student, Student Research Center, School of Dentistry, Shahid Beheshti University of Medical Sciences, Tehran, Iran.; 3 Division of Oral and Maxillofacial Pathology, Dept. of Diagnostic Sciences and Oral Medicine, University of Tennessee Health Science Center, College of Dentistry, Memphis, TN, USA.; 4 Iranian Center for Endodontic Research, Research Institute for Dental Sciences, Shahid Beheshti University of Medical Sciences, Tehran, Iran.

**Keywords:** Jaw Cysts, Jaw Neoplasms, Odontogenic Cysts, Odontogenic Tumors

## Abstract

**Background:**

Hybrid odontogenic lesions (HOLs) show combined microscopic features of two or more recognized odontogenic cysts and neoplasms, occurring in the same primary location. These lesions are uncommon and there is limited information on the clinical and microscopic features of such lesions.

**Purpose:**

We aimed to assess the frequency and types of HOLs admitted to a main oral pathology center in Iran in 30 years.

**Materials and Method:**

In this retrospective observational study, the archives of the Oral and Maxillofacial Pathology Department of Shahid Beheshti University of Medical Sciences from 1993 to 2022 were reviewed, and cases diagnosed with odontogenic lesions were selected. All microscopic slides were screened and cases of the HOLs were extracted.

**Results:**

Over 30 years, a total of 1767 cases (composed of 1264 cysts and 503 tumors) were found to be odontogenic lesions, of which 19 cases (1.07%) were classified as HOLs. The mean±SD and median age of patients were 22.57±13.19 and 15 years, respectively. The most common HOL was dentigerous cyst/odontoma (42.10%) followed by calcifying odontogenic cyst/odontoma (10.52%) and central odontogenic fibroma/central giant cell granuloma (10.52%). About 68.42% of the lesions were associated with impacted teeth. Radiographically, most of the HOLs had a mixed internal structure (68.42%) and were unilocular (73.68%). Most of the lesions showed painless expansion (63.15%). All cases were managed with surgical treatment alone, most of which had conservative surgery (enucleation of the lesion) (88.88%).

**Conclusion:**

HOLs are rare and show a wide variety of histopathologic features. HOLs generally showed the highest frequency in the second decade of life. Awareness of these microscopic patterns can lead to proper diagnosis and management.

## Introduction

The origin of odontogenic cysts is from the odontogenic epithelium entrapped in bone or gingiva, while odontogenic neoplasms are derived from the epithelial or ectomesenchymal components of the tooth-forming structures [ 1 ]. Hybrid odontogenic lesions (HOLs) show combined microscopic features of two or more recognized odontogenic cysts and neoplasms, occurring in the same primary location [ 1 - 2 ]. HOLs display various clinical features, ranging from asymptomatic to painful expansile lesions. Such pathological lesions must be carefully examined microscopically to identify the more aggressive and threatening compartment and to carry out an appropriate surgical approach based on the abovementioned compartment [ 3 ]. The most commonly reported HOLs include calcifying odontogenic cyst (COC)/odontoma, central odontogenic fibroma (COF)/central giant cell granuloma (CGCG), adenomatoid odontogenic tumor (AOT)/calcifying epithelial odontogenic tumor (CEOT), AOT/dentigerous cyst (DC) and DC/odontoma [ 1 ]. Several authors suggest that HOLs are not the result of a collision between two separate entities but also they probably develop from a common source or ameloblastomatous change in an existing odontogenic cyst due to the pluripotentiality of the odontogenic epithelium with both lesions probably developing [ 4 - 6 ]. This explanation may be correct for a subset of hybrid lesions, but there might be other cases that are composed of two separate entities [ 7 ]. HOLs are rarely reported in the jaws and may cause a diagnostic challenge for oral pathologists/clinicians because of their debatable histogenesis and not well-understood clinical behavior [ 1 , 7 ]. Treatment modalities are usually based on the component which is associated with a more aggressive course [ 7 ]. The purpose of this study was to assess the frequency and histopathologic types of HOLs in a main academic center of oral pathology service in Iran for 30 years.

## Materials and Method

This retrospective observational study was approved by the Ethics Committee of Shahid Beheshti University of Medical Sciences (SBMU) (IR.SBMU.DRC. REC.1401.019). In this cross-sectional study, 9013 biopsy reports released from the Oral and Maxillofacial Pathology Department of SBMU were reviewed from 1993 to 2022. Those cases with a diagnosis of odontogenic lesions were included in this study. The histopathologic slides were reviewed by two expert oral pathologists. Non-hybrid odontogenic lesions were excluded from the study. Then, the patient’s demographic information including age, and gender, and all clinicopathological information of the HOLs including location, size (<2 cm and ≥2 cm), clinical findings, radiographic features, histopathologic diagnosis, and treatment plan was recorded and categorized in the Excel tables. Then, a descriptive statistical analysis was performed using SPSS software (SPSS version 26.0; IBM, Armonk, NY, USA). 

## Results

Within the 30 years, a total of 1767 cases (1264 cysts and 503 tumors) had been diagnosed as odontogenic lesions, of which 19 cases (1.07%) were HOLs and included in this study. HOLs generally showed the highest frequency in the second decade of life (42.10%) with a mean age of 22.57±13.53 (ranging from 7 to 64 years). Fourteen patients were male (73.68%) and 5 cases were female (26.31%) (M/F= 2.8/1). The most commonly site was the posterior of the mandible (n=7, 36.84%) followed by the anterior of the mandible (n= 5, 26.31%). The most common diagnosed HOL was DC/odontoma (n=8, 42.10%) followed by COC/ odontoma (n=2, 10.52%) and COF/ CGCG (n=2, 10.52%). Rare variants such as glandular odontogenic cyst (GOC) with ameloblastoma (n=1), odontogenic keratocyst (OKC) with ameloblastoma (n=1), and unicystic ameloblastoma with ameloblastic fibroma (AF) (n=1) were also seen (Table1). Most of the HOLs were unilocular (73.68%) with mixed internal structure (68.42%).

**Table 1:** Histopathologic diagnosis and demographic information of hybrid lesions

Histopathologic diagnosis	N	Mean age (range)	Gender		Lesion location			
			Male	Female	Mandible	Maxilla	Anterior	Posterior
DC + odontoma	8	28.12 (14-64)	7	1	4	4	3	5
COF + CGCG	2	12.5 (10, 15)	-	2	-	2	2	-
COC + odontoma	2	16 (8, 24)	1	1	2	-	-	2
AOT+ odontoma	1	35	1	-	-	1	-	1
COC + AF	1	17	1	-	1	-	-	1
COC + Am	1	13	1		1	-	-	1
OKC + Am	1	21		1	1	-	1	-
GOC + Am	1	38	1		1	-	1	-
AF + Uni Am	1	7	1		1	-		1
AOT + DC	1	16	1	-	1	-	1	-
Total	19	23.3	14	5	12	7	8	11

DC: Dentigerous cyst; COF: Central odontogenic fibroma; CGCG: Central giant cell granuloma; AOT: Adenomatoid odontogenic tumor; COC: Calcifying odontogenic cyst; AF: Ameloblastic fibroma; Am: Ameloblastoma; OKC: Odontogenic keratocyst; GOC: Glandular odontogenic cyst; Uni Am: unicystic ameloblastoma

Most of the lesions demonstrated painless expansion (63.15%) and about 68.42% of them were associated with impacted teeth (Table 2). The implemented treatment plan for most of them (88.88%) was conservative surgery (Table 3).

**Table 2:** Radiographic information of the reviewed hybrid lesions

Histopathologic diagnosis	N	Internal structure		LocularityImpacted tooth		Root	resorption	Tooth displacement/ divergence	Mobility	Trismus
		Lucent	Mixed/ opaque	Uni	Multi					
DC + odontoma	8	-	8	8	-	8	1	-	1	1
COF + CGCG	2	-	2	1	1	2	-	1	-	-
COC + odontoma	2		2	2	-	-	-	-	-	-
AOT + odontoma	1		1	1	-	NA	NA	NA	-	-
COC + AF	1	1	-	NA	NA	NA	NA	NA	-	-
COC + Am	1	1	-	-	1	1	1	1	-	-
OKC + Am	1	1	-	1	-	-	-	1	-	-
GOC + Am	1	1	-		1	-	1	1	1	-
AF+Uni Am	1	1	-		1	1	-	-	-	-
AOT + DC	1	1		1	-	1	NA	NA	NA	-
Total	19	6	13	14	4	13	3	4	2	1

NA: Not available; Uni: Unilocular; Multi: Multilocular; DC: Dentigerous Cyst; COF: Central odontogenic fibroma; CGCG: Central giant cell granuloma; COC: Calcifying odontogenic cyst; AOT: Adenomatoid odontogenic tumor; AF: Ameloblastic fibroma; AM: Ameloblastoma; Uni Am: Unicystic ameloblastoma

**Table 3:** Clinical signs and symptoms, lesion size, and treatment modality of the reviewed hybrid lesions

Histopathologic diagnosis	N	Painless expansion	Painful expansion	Asymptomatic	Size of lesion		Treatment modality
					2>	2<	
DC + odontoma	8	3	2	3	5	3	Enucleation
COF + CGCG	2	2	-	-	2	-	Enucleation
COC + odontoma	2	1	-	1	2	-	Enucleation
AOT+ odontoma	1	1	-	-	-	1	Enucleation
COC + AF	1	1	-	-	NA		NA
COC + Am	1	1	-	-	-	1	en block resection
OKC + Am	1	-	1	-	1	-	Enucleation
GOC + Am	1	1	-	-	-	1	Enucleation
AF + Uni Am	1	1	-	-		1	en block resection
AOT + DC	1	1	-	-	1	-	Enucleation
Total	19	12	3	4	11	7	

CD: Dentigerous cyst; COF: Central odontogenic fibroma; CGCG: Central giant cell granuloma; COC: Calcifying odontogenic cyst; AOT: Adenomatoid odontogenic tumor; AF: Ameloblastic fibroma; Am: Ameloblastoma; OKC: Odontogenic keratocyst; Uni Am: Unicystic ameloblastoma

## Discussion

Collision and hybrid neoplasms indicate the occurrence of two or more separate synchronous benign or malignant primary tumors, appearing in the same anatomic area. Hybrid neoplasms are composed of two or more different tumoral entities in a single tumor that arise within a definite topographical region, while collision tumors are lesions that originate in different areas but combine in a specific region [ 8 ]. Yoon *et al*. [ 4 ] suggested that the biological mechanism causing these combinations is not easily defined. The possible pathogenic mechanisms are either the collision of two separate lesions or the transformation of one lesion into another lesion [ 4 ]. In this study, the overall frequency of HOLs accounted for 0.21% of all biopsies and 3.77% of the diagnosed odontogenic neoplasms. Neuman *et al*. [ 7 ] stated the percentage of HOL as 0.002% of all samples and likewise, Siar and Ng [ 9 ] found the frequency of HOLs to be 0.3% of odontogenic tumors. The present research showed a male predilection which is not consistent with previously reported findings [ 2 , 9 ]. In a recent systematic review, Pontes *et al*. [ 1 ] reported 24.5 years as the mean age of the cases with HOL, but in the other series, a lower mean age was described [ 9 ]. In the current study, there was a posterior mandibular predilection that was similar to other studies [ 1 , 7 ]. Interestingly, in most of our cases that were older than 30 years, the HOL had affected the anterior of the mandible (75%). Unlike the review of Pontes *et al*. [ 1 ], in the present investigation, 68.42% of lesions showed mixed radio-lucent/radio-opaque structures in radiographic views. This finding is probably related to a high number of odontomas as an associated lesion in our study. Moreover, due to the high rate of DC/odontoma in this observation, the number of unilocular lesions was higher, which is consistent with the findings of Pontes *et al*. [ 1 ]. In our research, the majority of the cases displayed painless expansion; although, asymptomatic lesions and painful expansion were also found which is in line with previous reports [ 1 , 9 ]. In the review performed by Pontes *et al*. [ 1 ], most cases were treated with enucleation. In the present study, about 88.88% were treated with conservative surgery and only two cases received en-block resection. Pontes *et al*. [ 1 ] reported recurrence in cases of COF/CGCG but unfortunately, follow-up information was not available in this study.

**Figure 1:**
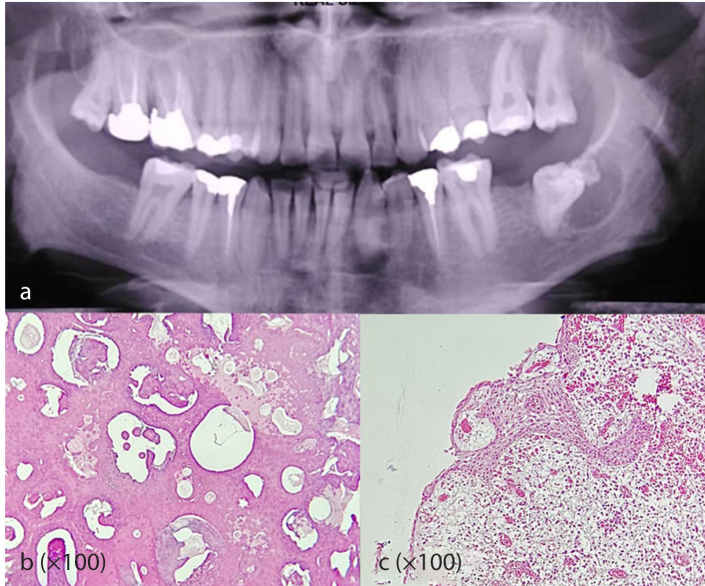
Dentigerous cyst/odontoma; **a:** The panoramic radiograph shows a well-defined corticated unilocular radiolucent lesion with radio-opaque mass around the crown of a semi-impacted left mandibular third molar; To prepare the sample, we had to separate the calcified part (odontoma) and put it in acid; thus, the images of the different parts of the lesion can be observed in two separate slides as follows, **b:** The microscopic section displays complex odontoma (Hematoxylin and eosin staining, 100×), **c:** Lining of the dentigerous cyst with nonkeratinized stratified squamous epithelium (Hematoxylin and eosin staining, 400×)

The most commonly reported HOLs in literature comprise COC/odontoma, COF/CGCG, AOT/CEOT, AOT/ DC, and DC/odontoma [ 1 ]. In the present study, DC/ odontoma was the most common HOL with a mean age of 28.12 and high male predilection (87.5%) (Figure 1). Additionally, the same distribution was seen in the mandible and maxilla. Although most of them were smaller than 2 cm, larger cases with trismus, tooth mobility, and root resorption were also reported. COC/ odontoma and COF/CGCG were in second place. In the review of COC in the literature, it has been emphasized that the above lesion may be associated with other odontogenic tumors, such as odontomas, AOTs, and ameloblastomas [ 10 ]. It should be noted that according to the fifth edition of the World Health Organization (WHO) classification of head and neck tumors (2022), COCs associated with odontoma are no longer separated from other COCs [ 11 ], but due to the consideration of this item as a HOL in previous studies [ 1 , 12 - 13 ], we also classified such lesions as a hybrid case. These two entities have been reported in several studies as the most prevalent examples of HOLs [ 1 , 12 ]. It has been reported that COC with odontoma occurs at a younger age with a mean age of 17 years [ 13 ]. In this regard, a recent systematic review indicated the average age of patients with compound odontoma associated with COC to be even lower, with an average age of 14.4 years, and in most cases, no recurrence was reported after enucleation [ 14 ]. The mean age of COC/odontoma in the present study also was about 16 years. Although most cases of COC were reported in the anterior region of the mandible [ 13 ]; the recent systematic review indicated the maxillary anterior region as the most common area for the cases of compound odontoma associated with COC [ 14 ]. However, both cases in our study affected the posterior mandible. Additionally, most of the reported HOLs composed of COC/AF were located in the posterior region of the mandible [ 15 ]. Furthermore, hybrid lesions of OKC/ odontoma have also been reported in the literature [ 3 ].

CTNNB1 gene (Wnt molecular pathway) is involved in COC pathogenesis. Furthermore, WNT/beta-catenin pathway activation in embryonic SOX-2 positive dental stem cells can induce odontoma formation [ 11 , 16 ]. This finding may explain the presence and concurrent development of COC/odontoma together.

Although CGCG is not an odontogenic lesion, based on the previous reports [ 1 , 13 ], we considered that in the hybrid category. COF/CGCG usually affects female patients and shows mandibular predilection with a mean age of 33 years [ 17 ]. In our study, both cases of COF/CGCG occurred in females and the anterior area of the maxilla with a mean age of 12.5 years. The pathogenesis and exact nature of hybrid COF/CGCG remain unclear and there are various concepts [ 17 ]. The first concept proposes the probability of being a "collision tumor/tumor-like" composed of a COF and CGCG arising in the same region [ 17 - 20 ]. Due to the rare nature of these neoplasms, this concept appears greatly unlikely [ 17 ]. It should be noted that recurrent cases of COF/ CGCG in most of the patients displayed features of both components of the hybrid lesion [ 17 - 19 ]. The second concept suggests that the growth factors, chemokines, and cytokines produced by the primary CGCG stimulate the proliferation of the odontogenic element and hence the formation of COF [ 19 , 21 ]. A third concept which seems more reasonable, states that COF is the primary neoplasm that induces a CGCG response to a trauma or other stimuli [ 18 - 20 ].

Ameloblastoma, as a benign epithelial odontogenic tumor with local aggressiveness, has many microscopic variations [ 22 ] and can be seen in combination with other lesions such as HOLs [ 23 ]. In this regard, rare lesions such as GOC/ameloblastoma, OKC/ ameloblastoma, and unicystic ameloblastoma/ AF were seen in our cases. The co-occurrence of OKC and ameloblastoma was first reported by Siar and Ng [ 24 ] under the name of keratoameloblastoma (KA). Whitt *et al*. [ 25 ] have divided KA into four broad groups: 

**A:** Papilliferous histology, in which the odontogenic epithelium has papillary projections into the cystic spaces. The Papilliferous nature of the epithelium appears to have arisen as a result of intercellular adherence and varying degrees of necrosis of individual cells. The necrotic cells separate from the remainder of the epithelium resulting in the formation of numerous pseudopapillary arrangements projecting into the lumen of the cystic follicles. 

**B:** Simple histology, in which epithelial follicles are lined by ameloblast-like cells with reverse polarity and filled with parakeratin or orthokeratin. 

**C:** Simple histology with OKC-like features that have similar patterns to simple type as well as features of conventional OKC.

**Figure 2:**
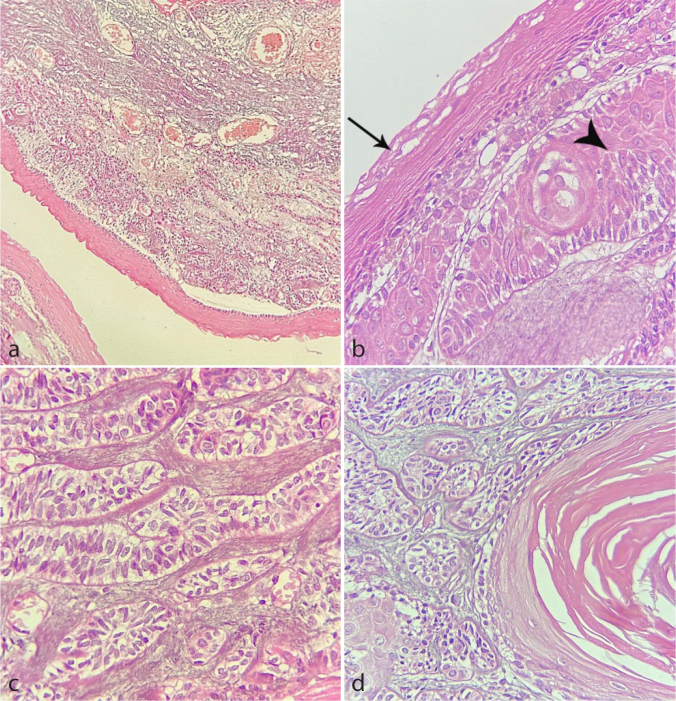
Histopathologic sections of odontogenic keratocyst/ameloblastoma, **a:** A cystic lesion lined by a parakeratinized stratified squamous epithelium with corrugated surface and palisaded basal cell layer (Hematoxylin and eosin staining, 100×), **b:** The cystic epithelium (arrow) and underlying ameloblastic nests with squamous metaplasia (arrow head) (Hematoxylin and eosin staining, 400×), **c-d:** The underlying connective tissue displays back to back ameloblastic islands with reverse polarity of peripheral cells (Hematoxylin and eosin staining, 400×, 100×; respectively)

**D:** Complex histology which is composed of epithelial follicles packed with parakeratin/orthokeratin and keratin masses extruded into connective tissue stroma in the form of Pacinian-like stacks with or without foreign body response. Therefore, the microscopic appearance of the present case (Figure 2) is similar to the case described by Neuman *et al*. [ 7 ], which showed a superficial OKC with palisading basal cell layer, corrugated surface, and islands of ameloblastoma in the underlying connective tissue showing reverse polarity and apical vacuolization.

PTCH1 gene is the most common genetic modification seen in OKC [ 11 ]. Interestingly, activating mutation in the BRAF p.V600E gene essentially is associated with ameloblastoma, but expression of its mutated protein product has not been described in OKC [ 26 - 27 ]. This finding may describe the presence of OKC/ ameloblastoma together.

**Figure 3:**
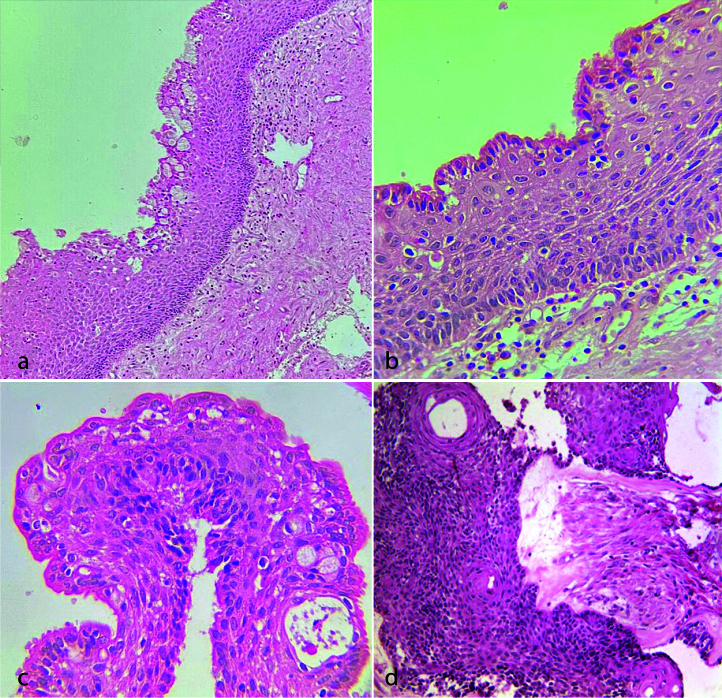
Histopathologic sections of glandular odontogenic cyst/ameloblastoma, **a:** A cystic lesion lined by hyperplastic stratified squamous epithelium containing numerous mucous cells (Hematoxylin and eosin staining, 100×), **b:** The surface hobnail appearance (Hematoxylin and eosin staining, 400×), **c:** A duct-like space (Hematoxylin and eosin staining, 400×), **d:** The ameloblastic proliferation (Hematoxylin and eosin staining, 100×)

A glandular odontogenic cyst associated with ameloblastoma is an exceptionally rare microscopic feature with no known clinical significance or treatment applications. This lesion is mostly reported in men, mandible, affecting younger patients with an average age of 20.8. GOC may show microscopic characteristics that overlap with botryoid odontogenic cysts, DC, and low-grade mucoepidermoid carcinoma, but not with ameloblastoma [ 28 ]. In the histopathologic features of our case presented here, glandular features including squamous epithelium with surface cuboidal to columnar cells, hobnail appearance, focal nodular thickening, mucin-producing goblet cells, and duct-like spaces were evident. Interestingly, in some areas, a reverse polarity of the basal cells was also noted and the underlying connective tissue demonstrated scattered islands with ameloblastomatous changes (Figure 3).

In the current study, there was a hybrid case of AOT/ DC in a 16-year-old boy in the anterior of the mandible. It should be noted that AOT infrequently shows complete cystic histopathologic features. Some authors have proposed the term “adenomatoid odontogenic cyst” (AOC) which seems to be a more appropriate term. They describe such lesion as a cyst with intraluminal proliferation, which fills the cystic space giving a solid appearance [ 29 ]. Several cases of de novo cystic AOT have been also described [ 29 - 31 ]. In the present study, several cases with a hybrid diagnosis were excluded because they seemed to be a cystic form of AOT rather than a hybrid lesion.

**Figure 4:**
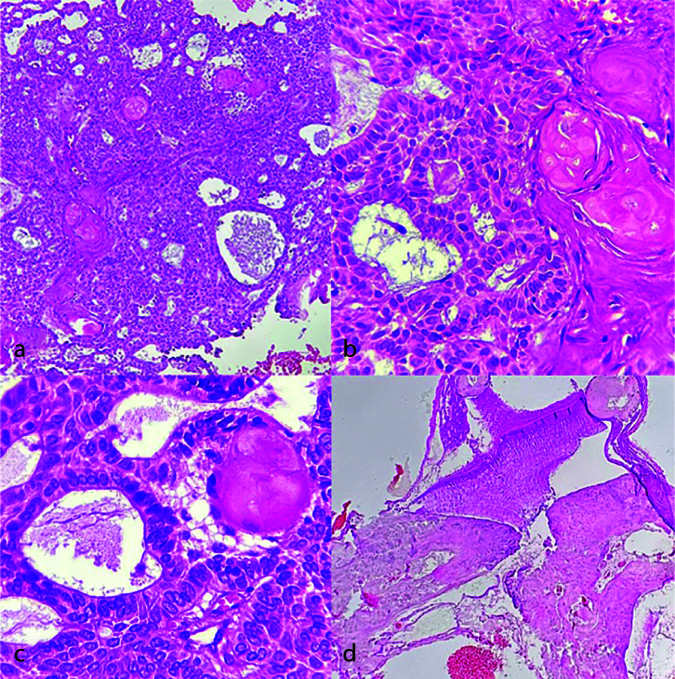
Histopathologic sections of adenomatoid odontogenic tumor with excessive ghost cells/odontoma, **a-b:** Many duct-like structures and ghost cells (Hematoxylin and eosin staining, 100×), **c:** Same image with higher magnification (Hematoxylin and eosin staining, 400×), **d:** The odontoma part (Hematoxylin and eosin staining, 100×)

In our research, there is a case of AOT with excessive ghost cell production and odontoma (Figure 4). Due to the absence of ameloblast-like cells with reverse polarity in the lesion, a diagnosis of dentinogenic ghost cell tumor (DGCT) could not be made for this hybrid lesion. Gomez *et al*. [ 32 ] described 24 years as the average age of this variant, and most of the cases showed a tendency to occur in the posterior region of the mandible. The presence of ghost cells in AOT has been also reported [ 33 ]. 

**Figure 5:**
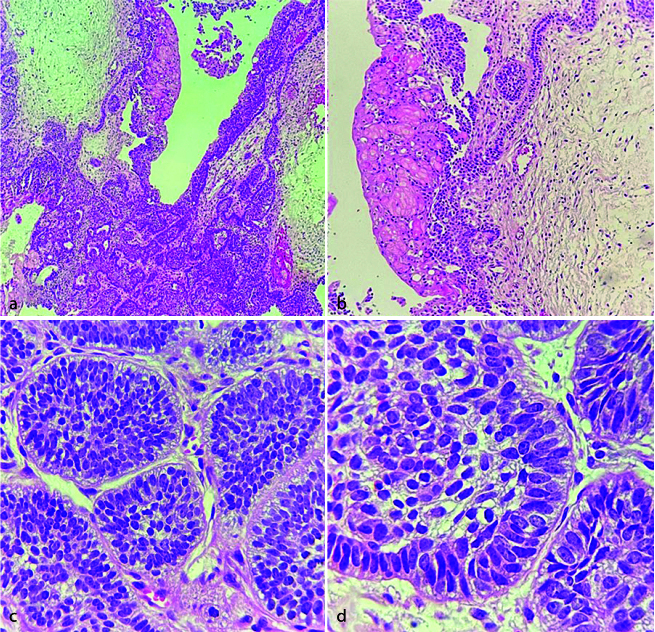
Histopathologic sections of ameloblastoma ex calcifying odontogenic cyst, **a:** A cystic lesion lined by ameloblastic epithelium contain ghost cells in which the underlying connective tissue demonstrates numerous ameloblastic islands in fibrous stroma (Hematoxylin and eosin staining, 4×), **b:** The lining of calcifying odontogenic cyst (Hematoxylin and eosin staining, 100×), **c-d:** The ameloblastic part (Hematoxylin and eosin staining, at magnification of 100× and 400×; respectively)

Several cases of COC in association with ameloblastoma have been reported, but there are confusing cases for this category. Epithelial proliferation in COC may mimic ameloblastoma [ 2 ]. In the histopathologic sections, “ameloblastomatous COC” is similar to a unicystic ameloblastoma; however, the presence of the ghost cells and calcifications within the proliferative epithelium can distinguish a COC from an ameloblastoma. Ameloblastomatous COC should be distinguished from “ameloblastoma ex COC”, as the former needs a conservative treatment and the latter requires aggressive management [ 34 ]. In our case, extensive proliferation of ameloblastomatous islands was seen in the connective tissue of the cyst, showing prominent hyperchromic basal cells with reverse polarity. Although some scattered ghost cells were seen in some islands, ameloblastomatous appearance was predominantly seen. In addition, the invasive behavior of the lesion, its large size (extending from the right mandibular first molar to the condyle), and the presence of root resorption suggested a diagnosis of ameloblastoma ex COC (Figure 5). 

**Figure 6:**
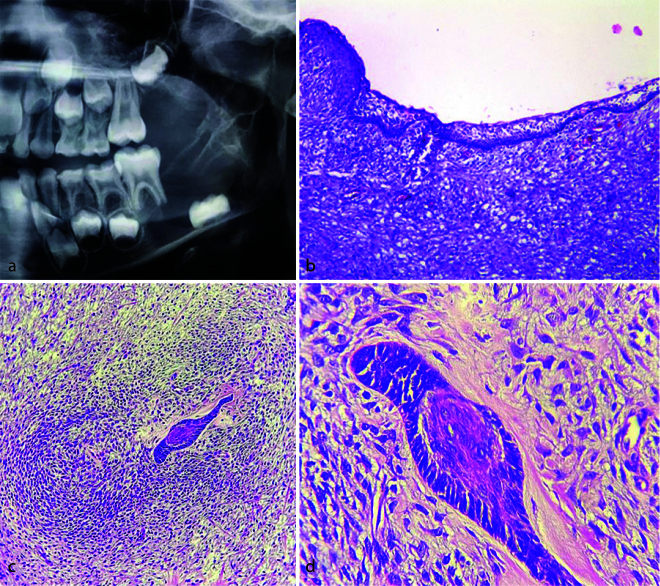
Radiographic view and histopathologic sections of ameloblastic fibroma/unicystic ameloblastoma, **a:** The panoramic radiograph reveals a well-defined corticated multilocular radiolucent lesion which involves the newly formed mandibular left second molar tooth bud, **b:** A cystic lesion lined by ameloblastic epithelium (Hematoxylin and eosin staining, 100×), **c-d:** The underlying connective tissue demonstrates dental papilla-like stroma containing scattered ameloblastic islands (Hematoxylin and eosin staining, at magnification of 100× and 400×; respectively)

COC would be the most commonly reported lesion in association with AF. However, unicystic ameloblastoma and cystic changes without prominent epithelial lining have been also described . Our case was a 7-year-old boy with a large expansion of the posterior mandible of short duration, who underwent en block resection. Microscopically, cystic epithelium similar to luminal ameloblastoma was seen, and the connective tissue showed dental papilla features, containing scattered islands with ameloblastomatous changes (Figure 6). BRAF p.V600E mutations commonly are identified in conventional and unicystic ameloblastoma. AFs also may show BRAF p.V600E mutations, which can explain the presence of these two lesions together [ 11 ]. 

In a previous study, Chatterjee *et al*. [ 38 ] described CTNNB1 mutation in several odontogenic lesions such as ameloblastoma, COC, DGCT, and malignant odontogenic tumors. They found beta-catenin as a useful diagnostic element involved in the pathogenesis of odontogenic lesions. This finding may describe the variety of HOLs. The major limitation of this study was the lack of follow-up information to determine any recurrence after treatment of the lesions, especially after conservative surgery. In addition, genetic mutations were not assessed in our cases. 

## Conclusion

HOLs are rare and show a wide variety of histopathologic features. The most common HOLs in a pathology center in Iran for 30 years were DC/odontoma. All mentioned lesions were managed with surgical treatment alone, most of them were removed by enucleation. Similar to other case series, the lesions tended to occur in younger age and mandible. Clinical symptoms varied from asymptomatic to painful or expansile lesions. Reporting more case series can lead to proper diagnosis and effective management. 
